# Thermal Healing, Reshaping and Ecofriendly Recycling of Epoxy Resin Crosslinked with Schiff Base of Vanillin and Hexane-1,6-Diamine

**DOI:** 10.3390/polym11020293

**Published:** 2019-02-10

**Authors:** Van-Dung Mai, Se-Ra Shin, Dai-Soo Lee, Ilho Kang

**Affiliations:** 1 Division of Semiconductor and Chemical Engineering, Chonbuk National University, Baekjedaero 567, Deokjin-gu, Jeonju, Chonbuk 54896, Korea; dungmv1983@gmail.com (V.-D.M.); srshin89@jbnu.ac.kr (S.-R.S.); 2 Research Center, NEPES AMC, 99 Seokam-ro, Iksan, Chonbuk 54587, Korea

**Keywords:** crosslinked epoxy resin, thermal-healing, recycling, imine metathesis, bio-derived hardener

## Abstract

A bio-derived dihydroxylimine hardener, Van2HMDA, for the curing of epoxy resin was prepared from vanillin (Van) and hexamethylene-1,6-diamine (HMDA) by Schiff base formation. The epoxy resin of diglycidyl ether of bisphenol A was cured with Van2HMDA in the presence of the catalyst, 2-ethyl-4-methylimidazole (EMI). The crosslinked epoxy resin showed thermal-healing properties at elevated temperatures. Moreover, the crosslinked epoxy resin can be reshaped by heating via imine metathesis of the hardener units. The imine metathesis of Van2HMDA was confirmed experimentally. Stress-relaxation properties of the epoxy resin crosslinked with Van2HMDA were investigated, and the activation energy obtained from Arrhenius plots of the relaxation times was 44 kJ/mol. The imine bonds in the epoxy polymer matrix did not undergo hydrolysis after immersing in water at room temperature for one week. However, in the presence of acid, the crosslinked polymer was easily decomposed due to the hydrolysis of imine bonds. The hydrolysis of imine bonds was used for the ecofriendly recycling of crosslinked polymer. It is inferred that thermal-healing, reshaping, and reprocessing properties can be implemented in the various crosslinked epoxy resins with the bio-derived dihydroxylimine hardener, albeit the recycled epoxy resin is of inevitably lower quality than the original material.

## 1. Introduction

Epoxy resin is a representative thermosetting polymer that has been widely used because of its excellent thermal and chemical stability and good mechanical properties. However, because, upon curing, epoxy resins form a highly crosslinked network structure, they cannot be reshaped or recycled at the end of their service life or after they have been damaged and may pose a risk to the environment and to human health. Therefore, extensive efforts have been devoted to designing thermosetting epoxy resins that can be reshaped or recycled and to extending the lifetimes of epoxy resins by introducing healing properties.

The first strategy to introduce healing properties into epoxy resins involves introducing containers or capsules that contain a healing agent [1,2,3]. In this method, when a crack is formed, the capsules are broken, thereby releasing the healing agent to heal the crack. This extrinsic healing strategy has limitations with respect to repeated healing capability, and the epoxy resin still cannot be reshaped or recycled after crosslinking. The second strategy for healing capability is the introduction of dynamic covalent bonds into the polymer matrix. This intrinsic healing can be classified into one of two types—dissociative or associative—based on the mechanism.

In the dissociative-type mechanism, chemical bonds are first broken upon stimulus and, then, formed again under a different stimulus. The typical dissociative-type mechanism is based on a Diels–Alder reaction [4,5,6,7]. Heating of products crosslinked by a Diels–Alder reaction induces a retro Diels–Alder reaction that accompanies the dissociation of the crosslinked structure and a decrease in viscosity that enables the damaged parts of the polymer to heal. However, upon cooling, the Diels–Alder reaction cannot proceed to completion and the polymer cannot be restored its original crosslinking density.

In the associative-type mechanism, dynamic covalent bonds are broken and are reformed at the same time. Because of the nature of the exchange reactions, the crosslink structures are maintained; however, the viscosity of the polymer can be controlled, enabling the epoxy thermoset to be processed like a thermoplastic [8,9,10,11,12,13,14,15,16,17,18,19,20] at elevated temperatures. The thermosetting epoxy resins containing such bonds have properties similar to those of a conventional thermoset at temperatures below a critical temperature (i.e., the vitrification temperature); however, at temperatures above this temperature, its behavior is similar to that of thermoplastics in general [8]. Examples of the exchange reactions include transesterification [8,9,10,11], metatheses of disulfides [12,13,14,15,16], olefins [17], boronic esters [18,19], and acylhydrazones [20]. Another type of linkage that can undergo exchange reactions is the C=N bond of an imine or Schiff base. The imine compounds can be easily synthesized by a condensation reaction between a primary amine and an aldehyde or ketone [21]. The reversible reaction of C=N includes hydrolysis (reaction with water), transamination (reaction with another primary amine), and imine metathesis (reaction with another imine bond). The mechanism of the imine exchange reaction in an organic solvent has been studied [22] which has revealed that imine metathesis proceeds faster under mild conditions in the presence of a small amount of primary amine as a catalyst. The imine bond has also been used to design polyimines with healing and recycling ability [23,24,25] and applied in self-assembly polymers [26,27,28]. The thermal-healing epoxy resin system based on imine metathesis has been reported [29]. However, this polymer shows low glass-transition temperature and low tensile strength in comparison with epoxy polymers composed of ester bonds [8] or disulfides bonds [12,13].

**Scheme 1:**
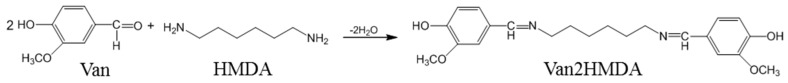
Condensation reaction of Van with hexane-1,6-diamine (HMDA) to prepare the Schiff base, Van2HMDA.

Concerning the environmental issue, petroleum-based materials should be replaced with bio-based materials from renewable resources. Among bio-based materials that can be incorporated into epoxy resin systems, vanillin (Van; 4-hydroxy-3-methoxybenzaldehyde) has been used to prepare epoxy resins [30,31,32]. Natural vanillin is one of the components extracted from vanilla orchid pods, which is widely used in food industry. However, the Van used in industries is not sourced natural vanillin to minimize the production cost as natural vanillin is expensive. Synthetic vanillin is industrially produced based on petroleum raw material and biomaterial such as wood. Among the biomaterials, lignin is a promising material [33] because this is a byproduct of the paper industry. In the present work, we present a simple route to prepare a bio-derived dynamic hardener (Scheme 1) for epoxy resin and study on its curing behavior as well as its thermal-healing, reshaping, and recycling abilities.

## 2. Materials and Methods

### 2.1. Materials

Hexane-1,6-diamine (HMDA), vanillin, 4-hydroxylbenzaldehyde (HBA), and 2-ethyl-4-methylimidazole (EMI) as a catalyst were purchased from Sigma-Aldrich (Yong-In, Korea). A diglycidyl ether of bisphenol A (DGEBA)-based epoxy resin with an epoxy equivalent weight of 187 g/eq (YD-128) was provided by Kukdo Chemical (Seoul, South Korea). Solvents used in this work include ethanol (EtOH), methanol (MeOH), toluene (Tol), dimethylformamide (DMF), and dimethylacetamide (DMAc), which were supplied by Samchun Pure Chemical (Pyeong-Taek, South Korea). All materials were used without further purification.

### 2.2. Preparation of Dihydroxylimine Compound

Van (30.43 g, 0.2 mol) was dissolved in 200 ml of EtOH followed by the addition of HMDA (11.62 g, 0.1 mol). The mixture was heated and was magnetically stirred at 80 °C for 12 h. A yellow precipitate was observed after the HMDA was dissolved in the Van solution. The precipitate, a dihydroxylimine compound (Van2HMDA), was collected by filtration, washed with cold EtOH and, then, was dried under vacuum at 70 °C for 24 h. The dried yellow powder (35.28 g, yield: 91.76%, mp=129–130 °C). ^1^H NMR (DMSO-d6, 600 MHz) chemical shift (δ, ppm): 8.10 (2H, –CH=N–), 7.27 (2H, Ar–H), 7.04 (2H, Ar–H), 6.77 (2H, Ar–H), 3.75 (6H, –OCH3), 3.44 (4H, –CH2– in hexane methylene), 1.54 (4H, –CH2– in hexane methylene), 1.30 (4H, –CH2– in hexane methylene). ^13^C NMR (DMSO-d6, 600 MHz) δ (ppm): 160.47 (–CH=N–), 148.44, 123.05, 115.72, 110.37 (C of Ar), 60.81 (–OCH3), 55.96 (–CH2–), 31.13, 27.15 (–CH2–).

The model compound based on HMDA and HBA, HBA2HMDA, was prepared via a similar process and was characterized by NMR analysis. ^1^H NMR (DMSO-d6, 600 MHz) δ (ppm): 8.11 (2H, –CH=N–), 7.48–7.50 (4H, Ar–H), 6.74–6.76 (4H, Ar–H), 3.41–3.43 (4H, –CH_2_– in hexane methylene), 1.51–1.53 (4H, –CH_2_– in hexane methylene), 1.27–1.29 (4H, –CH_2_– in hexane methylene). ^13^C NMR (DMSO-d6, 600 MHz) δ (ppm): 160.26 (–CH=N–), 130.00, 128.04, 115.92, (C of Ar), 60.94 (–CH_2_–), 31.18, 27.16 (–CH_2_–).

### 2.3. Curing of Bisphenol A-Based Epoxy Resin by Van2HMDA

A stoichiometric mixture of epoxy resin (YD-128, 10 g) and Van2HMDA (10.28 g) was heated and was mechanically stirred at 140 °C in a 250 mL reactor until the resin mixture was homogeneous. The resin was cooled to 100 °C followed by the addition of the EMI catalyst (1 wt %) under stirring for an additional 1 min. Then, the resin was cast and was cured at 120 °C for 24 h and, subsequently, post-cured further at 150 °C for 2 h to obtain a 1-mm-thick film.

### 2.4. Recycle of Crosslinked Epoxy Polymer Cured by Van2HMDA

The crosslinked epoxy polymer was recycled through the following procedure. Typically, 8 g of cured epoxy resin was added to 200 mL of 0.1 mol/L HCl standard solution. After the solution was heated and stirred at 70 °C for about 8 h, all polymer was dissolved. The solvent was removed by rotary evaporator at 65 °C. The hydrolyzed polymer was dried at 80 °C for 24 h and further 110 °C for 24 h to remove residue solvent and recover the polymer network by the condensation reaction.

### 2.5. Methods

The FT-IR spectra were recorded with a Jasco FT/IR 4100 (JASCO, Easton, MD, USA). The solid samples for FT-IR spectroscopy were mixed with KBr powder; then, they were ground, and pressed into thin tablets. The liquid samples were scanned after being coated directly onto KBr tablets.

The ^1^H NMR and ^13^C NMR spectra were collected with a 600 MHz FT-NMR spectrometer (JNM-ECA600, JEOL Ltd., Tokyo, Japan). The samples (~30 mg) were dissolved in 0.75 mL of dimethyl sulfoxide d_6_ (DMSO-d6).

Differential scanning calorimetry (TA Instruments DSC Q20, New Castle, DE, USA) was used to characterize the imine-compound-induced curing behavior of the epoxy resin. Dynamic mechanical analysis (DMA) was carried out on a TA Instruments DMA Q800 dynamic mechanical analyzer. The single-cantilever beam was used to deform the rectangular specimens with mean dimensions of 12.5 × 1 × 17.5 mm^3^. The frequency of the oscillation test was 1 Hz, and the temperature dependence of the behavior was investigated in the temperature range from 20 to 200 °C at a heating rate of 3 °C/min. In addition, stress-relaxation experiments were performed on the same mechanical analyzer. To maintain the straightness of the samples, initially, they were preloaded under a force of 0.001 N. The samples were heated to the test temperature and were held there for 30 min to reach thermal equilibrium. Then, 1% strain was applied, and the stress needed to maintain constant 1% strain was recorded. The stress-relaxation moduli were obtained as a function of time.

The imine metathesis was studied by liquid chromatography–mass spectrometry (LC/MS) (AGILENT 6410B, Agilent Technologies, Wilmington, NC, USA). The equivalent molar ratio of imine compounds Van2HMDA and HBA2HMDA were dissolved separately in MeOH, and the two solutions were mixed and stirred at room temperature. After one hour, the solvent was removed by vacuum evaporation at room temperature for the characterization.

The thermal healable of dynamic epoxy polymer was investigated by scratching test and was observed by scanning electron microscope (SEM) AIS2100C (Seron Technology, Ui-Wang, Korea). The surface of the film was coated with gold before observation. The surface of polymer was observed before scratch and after heating at 90 °C for 5 min.

Thermal stability of the epoxy polymer was analyzed by thermogravimetric analyzer (TGA, Instrument TA Q50, New Castle, DE, USA). Samples (10–20 mg) were loaded in a platinum pan and heated from 30 to 800 °C under a nitrogen at a heating rate of 20 °C/min.

Tensile test was carried out by Universal Test Machines (LR Plus series, LLOYD Instrument, AMETEK, West Sussex, UK). The epoxy polymer was casted in a dog-bone-shaped mold. The specimens were loaded to failure at a speed of 0.5 mm/min.

The gel fraction test was conducted by immersing crosslinked epoxy samples in different solvents at room temperature under static conditions. After a week, the solvents were removed by filtration and the samples were dried at 110 °C for 24 h. The gel fraction (GF) was calculated using the equation *GF* = *W*_2_/*W*_1_ × 100, where *W*_2_ is the weight of the dry sample and *W*_1_ is the weight of the original sample.

## 3. Results and Discussion

### 3.1. Preparation of Dihydroxylimine Compound

The imine was formed by a reversible condensation reaction between primary amine and carbonyl compounds. To enhance the yield of the reaction, benzene [34] were generally used as solvents to enable the removal of water formed by the condensation reaction by azeotropic distillation or by the addition of dehydrating agents. However, in this work, we found that the use of EtOH led to the imine compounds forming and precipitating out of the solution during the condensation reaction because of its low solubility in the reaction medium. Therefore, the condensation reaction is favorable for achieving a high product yield.

As shown in Figure S1a, the successful preparation of Van2HMDA was first confirmed by FT-IR analysis. The peak at 1673 cm^−1^ corresponding to a C=O stretching vibration (black curve in Figure S1a) of the aldehyde carbonyl group of Van and the peak between 3400 and 3500 cm^−1^ corresponding to NH stretching (red curve in Figure S1a) of the primary amine (HMDA) disappeared after the formation of Van2HMDA. The peak at approximately 1640 cm^−1^ corresponding to C=N stretching (Figure S1a, blue curve) of the imine group was clearly observed in the FT-IR spectrum of Van2HMDA. The broad peak at 3400 cm^−1^ corresponding to OH stretching vibration of hydroxyl group of Van (black curve in Figure S1a) also appeared on FT-IR curve of Van2HMDA (Figure S1a, blue curve), confirming its presence. Figure S1b shows the DSC curves of dihydroxylimine compound. The unique endothermal peak in the first heating curve (Figure S1b, black curve) with melting temperature around 129 °C indicating homogeneous crystal structure and high purification of Van2HMDA. However, no exothermal peak upon cooling and no endothermal peak upon second heating (red curve and blue curve in Figure S1b) was observed, indicating the slow crystallization of Van2HMDA.

The dihydroxylimine compounds were further characterized by ^1^H NMR and ^13^C NMR. Figure S2 shows the corresponding spectra. The formation of an imine group was confirmed by the peak at 8.1 ppm in the ^1^H NMR spectrum, corresponding to the proton of an imine group (Figure S2a). The carbon of the imine group was also confirmed by the peak at ~160 ppm in the ^13^C NMR spectrum (Figure S2b). The model compound, HBA2HMDA (4,4′-((hexane-1,6-diylbis(azanylylidene))bis(methanylylidene))diphenol) was also characterized by ^1^H NMR and ^13^C NMR as shown in Figure S3.

### 3.2. Imine Metathesis of the Model Compounds

**Figure 1:**
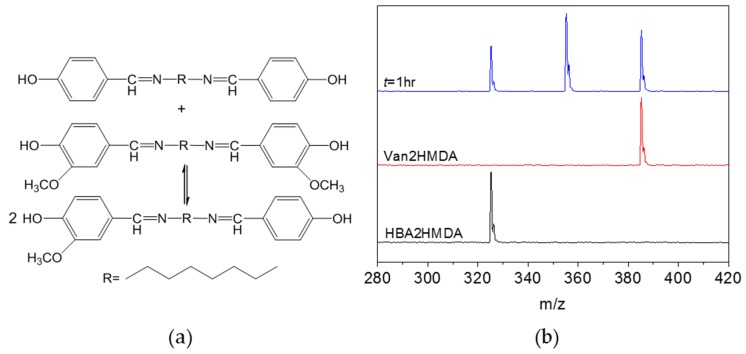
(**a**) Imine metathesis reaction between two imine compounds (Van2HMDA and HBA2HMDA) and (**b**) LC/MS spectra of imine compounds based on HBA2HMDA, Van2HMDA, and a mixture containing equivalent amounts of the two imine compounds in MeOH after 1 h (*t* = 1 h).

The imine C=N bond can undergo three different exchange reactions: hydrolysis, transamination, and imine metathesis. The imine metathesis can be accelerated by an organic base catalyst such as l-proline [35]. However, the presence of a small amount of primary amine can also accelerate imine metathesis [22,24]. The mechanism of imine metathesis with the formation of cyclic four-membered transition states has also been studied [22,24,25]. In this work the imine metathesis was again confirmed experimentally with the model molecules as shown in Figure 1. Two imine compounds with and without a methoxy group on their aromatic ring and, as a result, the metathesis product from two imine compounds of different m/z values were observed in the LC/MS spectrum after solutions of the two imine compounds were mixed. The new peak between the two starting peaks corresponds to the product of the imine metathesis reaction (blue line in Figure 1b).

### 3.3. Curing of Epoxy Resin by the Dihydroxylimine Compound

**Figure 2:**
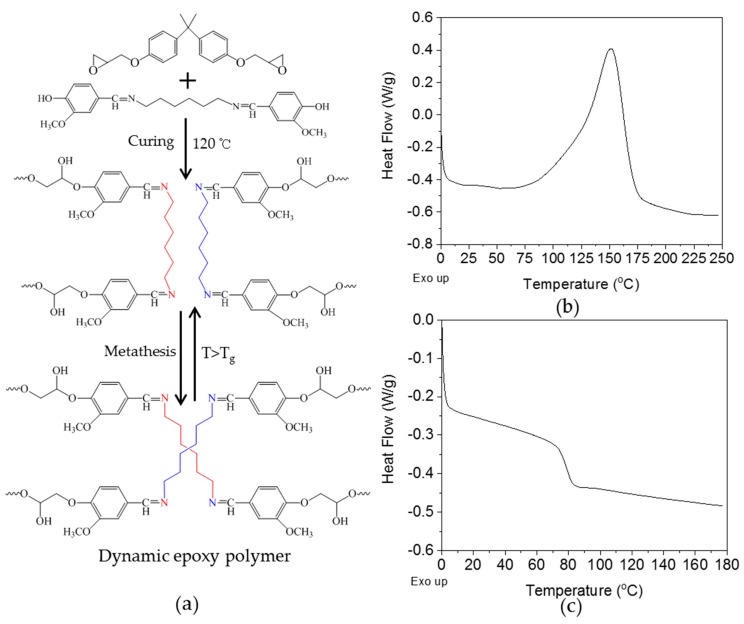
(**a**) Curing of bisphenol A-based epoxy resin by the dynamic hardener, Van2HMDA, and DSC thermograms of YD-128 cured by Van2HMDA: (**b**) 1st scan and (**c**) 2nd scan.

Epoxy groups in the resins can react with amines, hydroxyls, anhydrides, acids or thiols [36,37,38,39]. The amines can directly react with an epoxy group in the absence of a catalyst or initiator, whereas other hardeners generally react with an epoxy group with a catalyst or initiator present. In this study, EMI-initiated reactions of the hydroxyl groups of the dynamic hardener (Figure 2a) and epoxy groups were carried out. The exothermic peak in the first scan DSC thermogram in Figure 2b indicates an exothermic curing reaction between the hardener and the epoxy resin. The heat of cure was 133.6 J/g. In the second scan (Figure 2c), no exothermic peak was observed, indicating complete crosslinking of the epoxy resin after the first scan and a glass-transition temperature (*T*_g_) of 78 °C for the crosslinked polymer, as determined from the enthalpy change.

**Figure 3:**
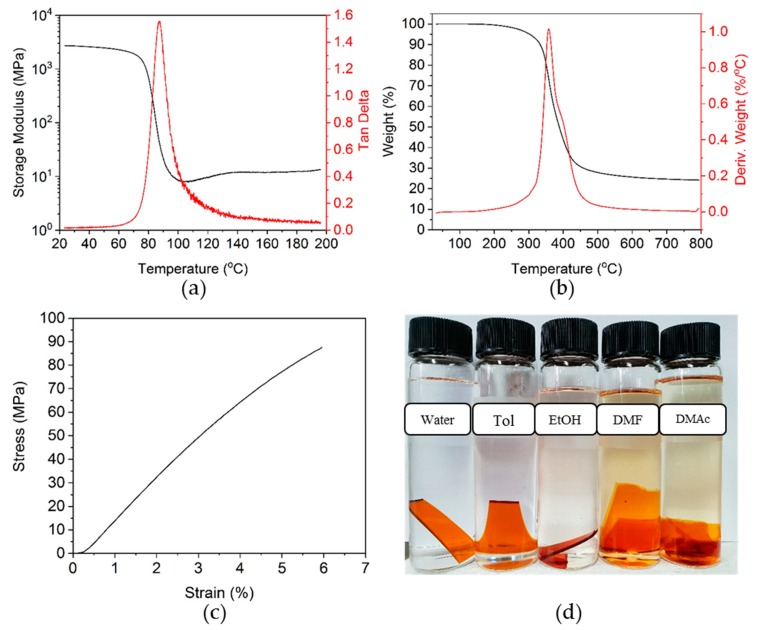
(**a**) DMA thermograms, (**b**) TGA thermograms, (**c**) Stress-strain behavior in tensile test, and (**d**) photographs of samples after a week of immersion in different solvents.

Figure 3a shows the storage modulus and tan delta of the epoxy resin cured by the dynamic hardener (Van2HMDA). A rubbery plateau is observed, and no melt flow zone appeared at temperatures up to 200 °C, indicating the crosslinked networks of epoxy polymer cured by dynamic imine hardener. The *T*_g_ identified from the tan delta of the epoxy cured by Van2HMDA was 87 °C, which is relatively low because of the inclusion of a flexible segment of hexamethylene in the dynamic hardener. Figure 3b shows the thermal stability of dynamic epoxy polymer. The onset degradation temperature (temperature at 5 wt % loss) of dynamic epoxy polymer was 302.9 °C. In comparisons with conventional epoxy polymer, this temperature was lower due to low bonding energy of imine bond. The crosslinked epoxy resin exhibited tensile strength of 85 MPa at elongation of 5.9 % (Figure 3c). These mechanical properties were superior to those of dynamic polymers based on the transesterification [8] or disulfide metathesis [13].

The GF test is a typical test to compare the crosslink density of thermoset polymers. The un-crosslinked polymer is dissolved in commonly used solvents. Results of the GF indicate that the epoxy resin crosslinked with dynamic polymer did not dissolve in common solvents (Figure 3d). In polar solvents such as DMF and DMAc, the polymer swelled, followed by partial dissolution of the low-molecular-weight polymer, resulting in a lower GF compared with those of the samples immersed in nonpolar solvents (Figure S4). The imine bonds can undergo hydrolysis to release the aldehyde and amine. However, after 1 week of immersion in water, the weight and the shape of the dynamic epoxy film showed almost no change, indicating that hydrolysis reaction hardly occurred. The highly crosslinked network may prevent the hydrolysis reaction. However, the crosslinked epoxy polymer was easily dissolved in acid solution due to the hydrolysis of imine bonds with acid catalyst. Therefore, crosslinked epoxy polymer can be ecofriendly recycled.

### 3.4. Stress Relaxation of Crosslinked Epoxy Resin

**Figure 4:**
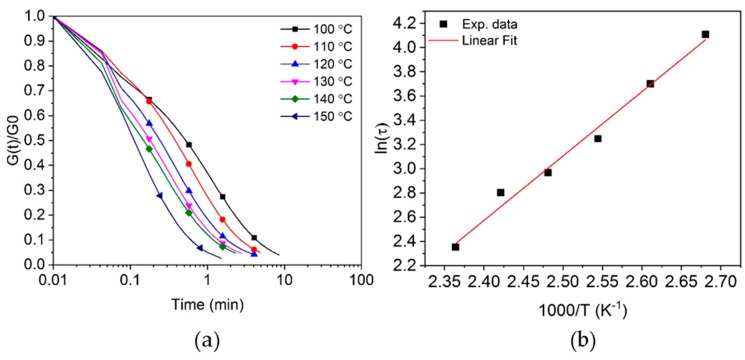
(**a**) Normalized stress-relaxation curves of the DGEBA-based epoxy resin cured by Van2HMDA at different temperatures; (**b**) Arrhenius plot of the relaxation times for the epoxy resins cured with Van2HMDA (*R*^2^ = 0.987).

Polymers containing dynamic bonds can relax stress and flow [8], which is a typical behavior of polymers with dynamic covalent bonds. The stress-relaxation behavior of the epoxy resin cured by the dynamic hardener were analyzed by DMA and shown in Figure 4a. The results showed that the epoxy resin crosslinked with Van2HMDA was able to fully relax stress at a temperature greater than the *T*_g_. Furthermore, the stress-relaxation curves shifted to the left with increasing temperature because of the increase of the imine metathesis rate, enabling stress to relax more quickly. Relaxation times (τ*) were defined based on Maxwell’s model for viscoelastic materials as the time required to relax to 1/e of the initial stress. The dynamic-hardener-based crosslinked epoxy resin in this study showed relaxation times that ranged from 60 s at 100 °C to 10 s at 150 °C, which are much shorter than those of the crosslinked epoxy resins cured by fatty acids [8] or disulfide content hardeners [12,13] reported in the literature indicating fast imine metathesis and rapid stress relaxation. The relationship between relaxation times and temperature according Arrhenius’ law is given as follows:

(1) $$\tau \left(T\right)=\tau_{0}exp\left(\frac{E_{a}}{RT}\right)$$

where *τ*_0_ is the relaxation time at infinite *T*, *E*_a_ is the activation energy of the imine metathesis, and *R* is the universal gas constant. Arrhenius plot of the relaxation time *τ* is shown in Figure 4b. From this fitting, an activation energy (*E*_a_) of 44 kJ/mol was obtained for the dynamic hardener crosslinked network of epoxy resin. This activation energy is much lower than the value of the crosslinked epoxy resins cured by fatty acids [8] or disulfide-based dynamic hardeners [12,13] reported previously.

The topology freezing transition temperature (*T*_v_) is a key transition temperature for a dynamic polymer because, at the *T*_v_, the polymer transforms from vitrified glass to deformable solid as a consequence of the exchange reactions in the network. This transition is identified when the viscosity increases to greater than 10^12^ Pa·s [40]. From the Maxwell relation, *η* = *G* · τ*, where *G* is calculated from the elastic modulus, as measured by DMA, according to the relation *G* = *E’*/2(1 + ν) (ν = 0.5, the Poisson’s ratio usually used for rubbers), where *G* = *E’*/3 and *E’* = 11.56 MPa; thus, τ* = 2.6 × 10^5^ s. Substituting τ* into the Arrhenius’s equation, we can get the *T*_v_. For the epoxy resin cured by Van2HMDA, the hypothetical *T*_v_ value was −38 °C, which is lower than its *T*_g_ (87°C, as determined by DMA), implying that an intrinsically fast imine metathesis occurred in a rigid polymer matrix. The value of *T*_v_ was calculated via extrapolation of stress-relaxation times; however, this transition is hypothetical because the network is not ultimately frozen by the reaction kinetics but by the lack of segmental motions associated with the *T*_g_ [41].

### 3.5. Thermal-Healing Properties and Reshaping Ability of the Crosslinked Epoxy Resin

**Figure 5:**
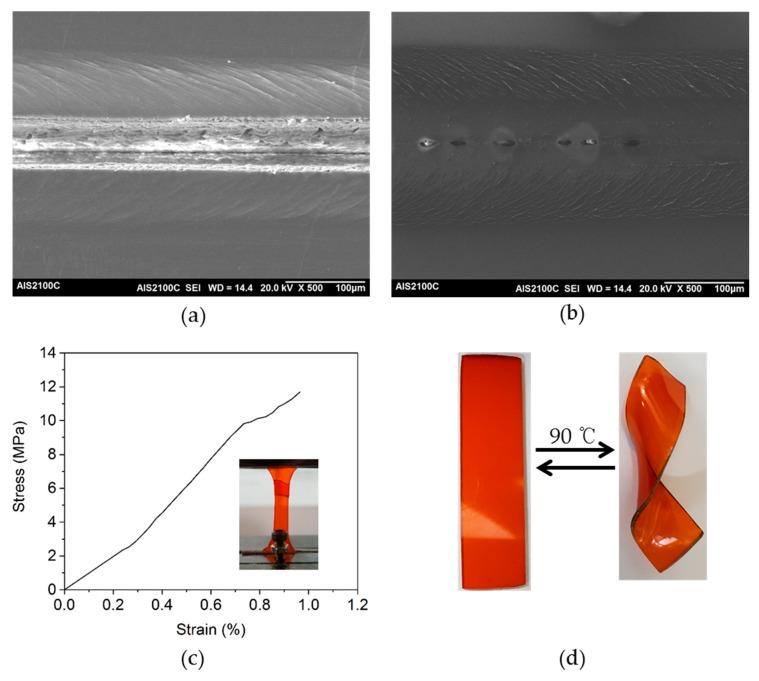
SEM images of (**a**) scratching surface and (**b**) the surface after heating at 90 °C for 5 min; (**c**) stress-strain behavior in tensile test of rebounded specimen by overlapping and heating at 110 °C for 15 min; (**d**) pictures showing the reshaping ability.

The thermal-healing tests were carried out by scratching of the surface of the films with a razor blade. The damaged film was heated on a hot plate at 90 °C for 5 min. Figure 5a shows the surface of polymer with a scratch 50 μm in width. After heating at 90 °C for 5 min, the scratch almost disappeared (Figure 5b). This temperature was higher than *T*_g_ therefore segmental motion was initiated that enables the polymer chains to diffuse into each other and the exchange reaction to occur. It is postulated that the healing process may start at the bottom of the scratch, where the distance between polymer molecules is sufficiently small for their chains to diffuse into each other.

In addition, we tested the healing property of the crosslinked epoxy resin by overlapping two pieces of specimen after tensile test between two glass plates, pressing them with a paper clip and, then, heating at 110 °C for 15 min (insect picture in Figure 5c). In this case, the crosslinked polymer molecules on the surface of the two pieces contacted each other because of stress. At high temperatures, the imine metathesis reaction occurred between these molecules, resulting in the healing of the two pieces. The tensile strength of healing sample was 10 MPa (Figure 5c), which was much lower than that of original sample because the failure occurred at healing area.

We confirmed the reshaping ability of the epoxy resin by heating a rectangular specimen to 90 °C on a hotplate and then twisting it. After the specimen was cooled to room temperature, it maintained its twisted shape (Figure 5d). The original shape of the film was recovered when the film was re-heated and applied the force. At temperatures greater than the *T*_g_, fast imine metathesis enables a change in the topology of the network and, upon cooling, the imine metathesis process becomes so slow that the topology of the network is essentially fixed [8].

### 3.6. Ecofriendly Recycling of Crosslinked Epoxy Polymer

Due to the crosslinked network structures, the thermosetting polymer cannot be reprocessed or recycled. Introduction of dynamic covalent bonds for the reprocessing or recycling of network polymers requires harsh conditions [12,13,23,24,25,29,42] such as high temperature, high pressure, additional monomer, and/or special catalyst. The imine bond can undergo three kinds of reversible reaction include hydrolysis (dissociative-type mechanism), transamination, and imine metathesis (associative-type mechanism). As shown above, imine metathesis could be used for thermal-healing and reshaping ability of crosslinked epoxy polymer. Then the hydrolysis of imine bond also could be used for ecofriendly recycling of crosslinked epoxy polymer.

**Figure 6:**
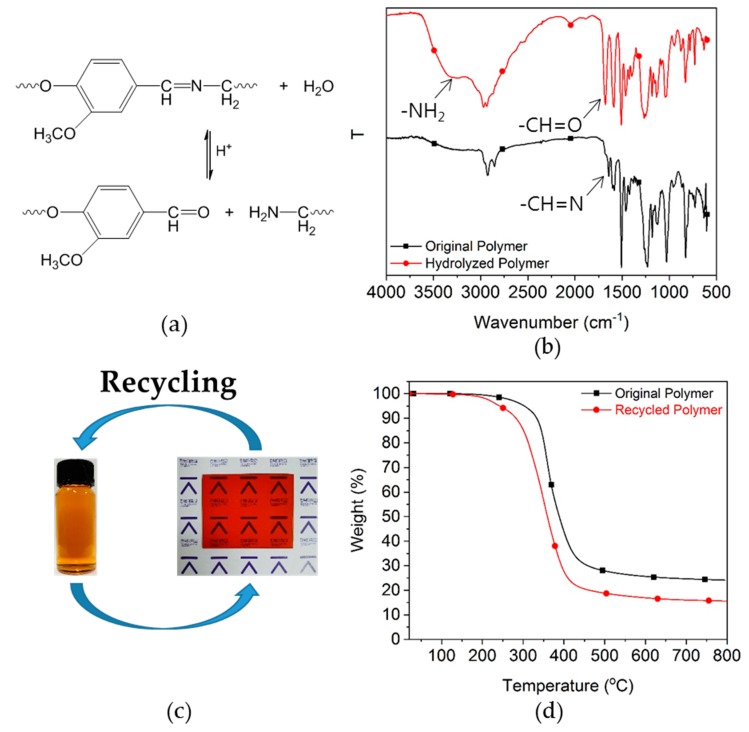
Recycling of epoxy resin crosslinked with Van2HMDA: (**a**) Hydrolysis of imine bonds; (**b**) FT-IR spectra of original polymer and hydrolyzed polymer; (**c**) Photographs show recyclability of the crosslinked epoxy resin; (**d**) TGA thermograms of original polymer and recycled polymer.

Figure 6 shows recyclability or reprocessing of epoxy resin cured by Van2HMDA. The hydrolysis of imine bonds was confirmed by FT-IR (Figure 6b). After hydrolysis, the peak at 1643 cm^−1^ (Figure 6b, black curve) characterizing for imine bond of original polymer was disappeared in FT-IR spectrum of hydrolyzed polymer (Figure 6b, red curve). The reformation of aldehyde and primary amine were confirmed by presence of peaks at 1677 cm^−1^ and 3323 cm^−1^ (Figure 6b, red curve). However thermal stability of recycled polymer was inferior to that of original polymer (Figure 6d). The onset degradation temperature of the recycled polymer was 242 °C, which is much lower than that of original polymer (302 °C). The glass-transition temperature (*T*_g_) of recycled polymer (63 °C) was lower than that of original polymer (78 °C) as given in Figure S5a. It is speculated that the condensation reaction between aldehyde and primary amine groups of the oligomers after chain scission via the hydrolysis of imine bonds could not be complete. Therefore, the crosslinking density of recycled polymer was lower than that of original polymer and the *T*_g_ was lowered. However, the recycled polymer still showed the reshaping and thermal-healing properties (Figure S5b,c).

## 4. Conclusions

In this study, we have shown a simple, environmentally friendly route to preparing a bio-derived dynamic hardener based on Van. The successful preparation of the dynamic hardener was confirmed by FT-IR and NMR analyses. The LC/MS spectra indicated imine metathesis of the model compounds. The dihydroxylimine compound can cure epoxy resin in a manner similar to the conventional hardener. The epoxy resin cured with a dynamic hardener exhibited fast stress relaxation because of rapid imine metathesis at temperatures greater than *T*_g_, enabling a dynamic crosslinked polymer with reshaping ability and thermal-healing ability. The dynamic epoxy polymer also shows ecofriendly recycle ability by using mild acid solution through hydrolysis of imine bonds. The recycled polymer shows lower thermal properties than that of original polymer due to lower crosslinking density. However, the recycled polymer still exhibits reshaping and thermal-healing ability.

## Supplementary Materials

The following are available online at https://www.mdpi.com/2073-4360/11/2/293/s1, Figure S1: (a) FT-IR spectra of Vanillin, HMDA, and the product (Van2HMDA) by Schiff-based formation and (b) DSC thermograms of Van2HMDA; Figure S2: (a) 1H-NMR and (b) 13C-NMR spectra of Van2HMDA based on Van and HMDA; Figure S3: (a) 1H-NMR and (b) 13C-NMR spectra of model compound HBA2HMDA based on HBA and HMDA; Figure S4: Gel fraction of the epoxy resin cured with Van2HMDA in different solvents; Figure S5: (a) DSC thermalgrams of original polymer and recycled polymer, (b) reshaping ability and (c) thermal-healing ability of recycled polymer.
