# Supplementary material for: Thermal Healing, Reshaping and Ecofriendly Recycling of Epoxy Resin Crosslinked with Schiff Base of Vanillin and Hexane-1,6-Diamine
Source: Polymers (Basel). 2019 Feb 10;11(2):293. doi: 10.3390/polym11020293 (PMC6419216; doi:10.3390/polym11020293)
Supplement: Supplementary file 1 [file polymers-11-00293-s001.pdf]

polymers

Supplementary Materials

Thermally healing, reshaping and ecofriendly recycling of epoxy resin crosslinked with Schiff-base of vanillin and hexane-1,6-diamine

Van-Dung Mai [1,†] , Se-Ra Shin[1,†] , Dai-Soo Lee [1,*], and Ilho Kang [2,*]

[1] Division of Semiconductor and Chemical Engineering, Chonbuk National University, Baekjedaero 567, Deokjin-gu, Jeonju, Chonbuk, 54896, Korea; dungmv1983@gmail.com(D.M.); srshin89@jbnu.ac.kr (S.-R.S.); daisoolee@jbnu.ac.kr(D.L.)

[2] Research Center, NEPES AMC, 99 Seokam-ro, Iksan, Chonbuk, 54587, Korea; kangilho@nepesamc.co.kr

* Correspondence: daisoolee@jbnu.ac.kr; Tel.: +82-63-270-2310; kangilho@nepesamc.co.kr; +82-63-833-2020

Received: date; Accepted: date; Published: date

† These authors contributed equally to this work.

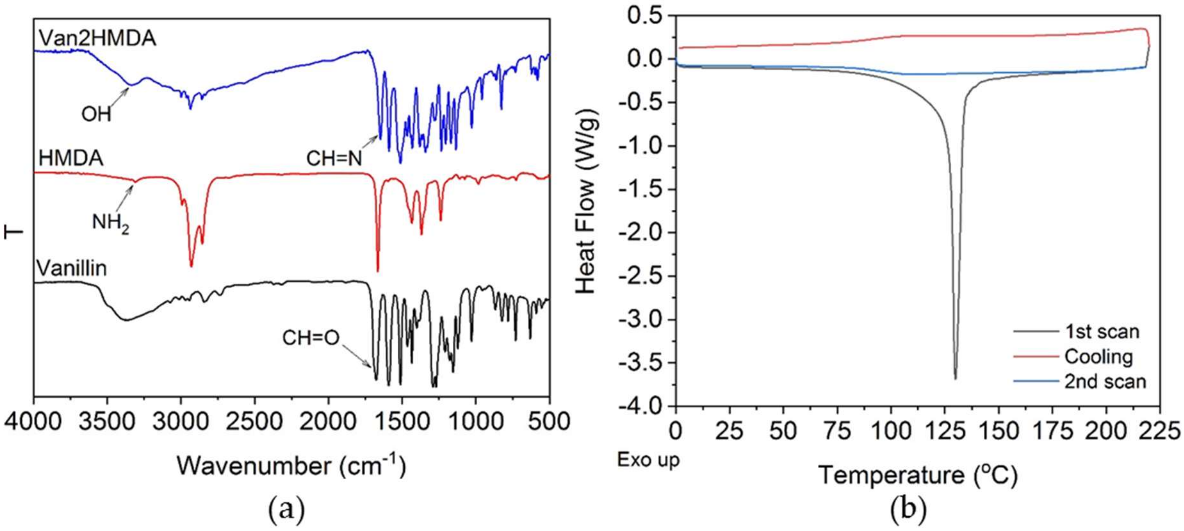

Figure S1. (a) FT-IR spectra of Vanillin, HMDA, and the product (Van2HMDA) by Schiff-based formation and (b) DSC thermograms of Van2HMDA.

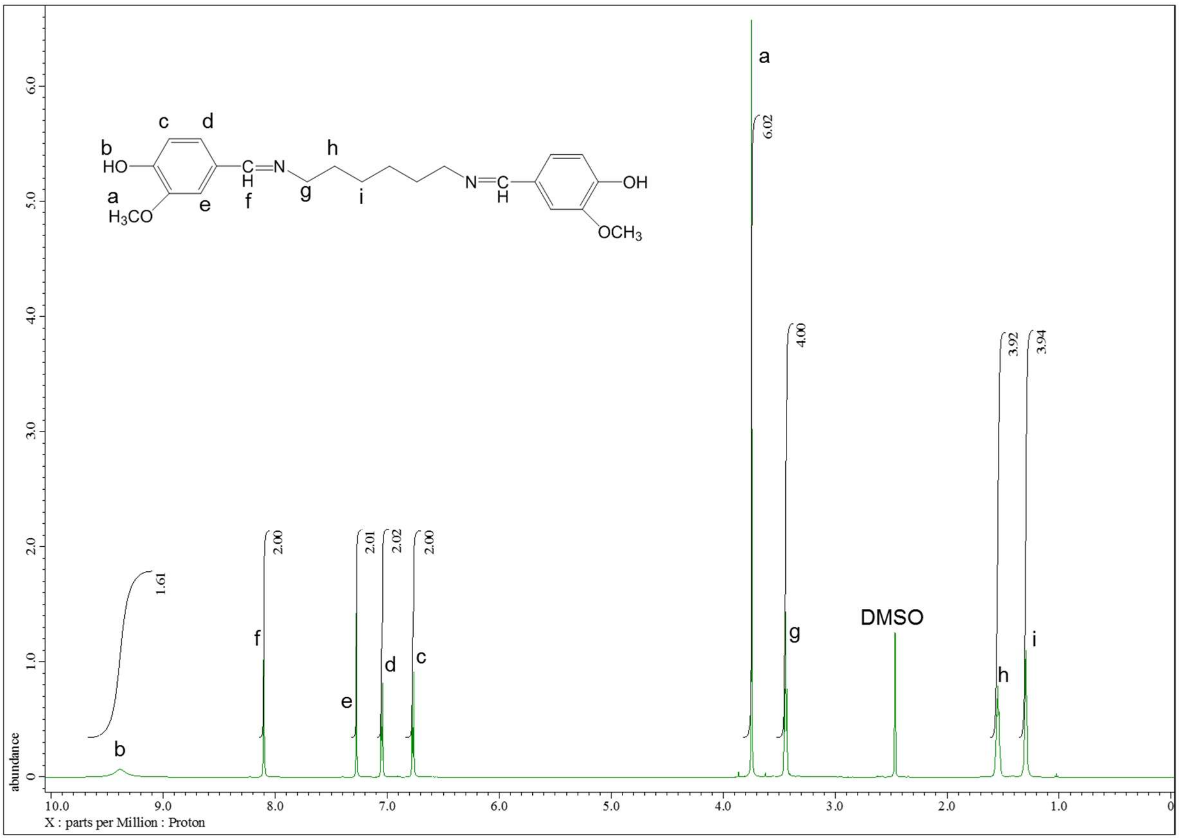

(a)

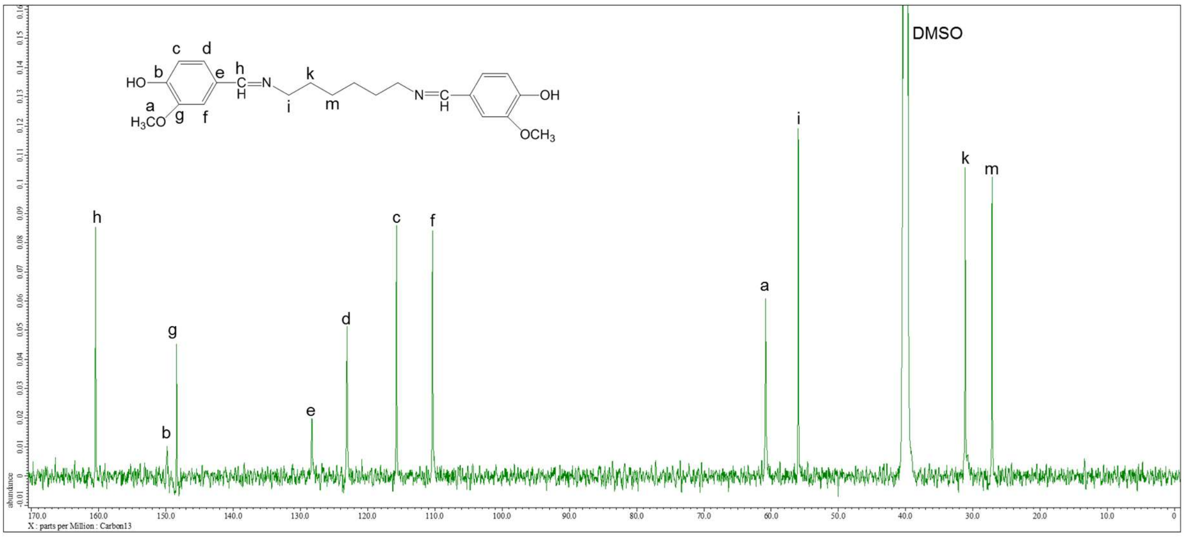

(b)

Figure S2. (a) ^{1}H-NMR and (b) ^{13}C-NMR spectra of Van2HMDA based on Van and HMDA.

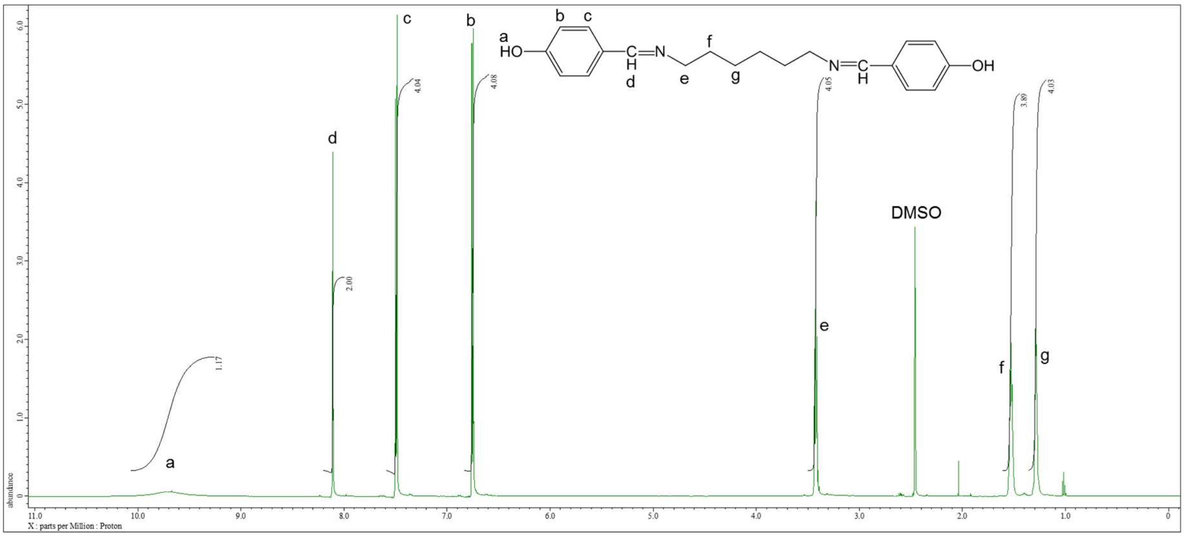

(a)

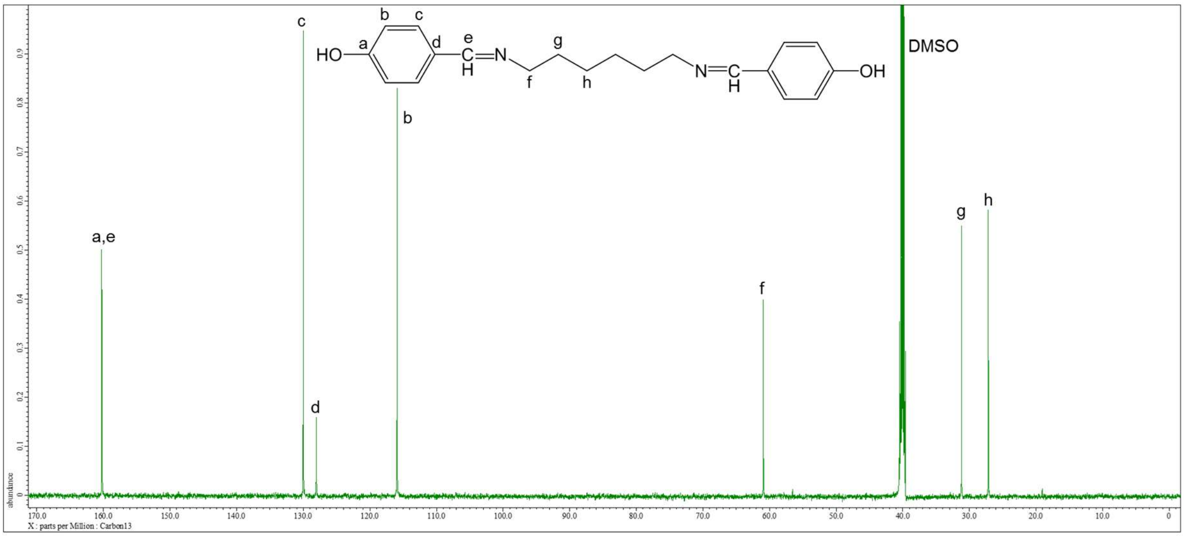

(b)

Figure S3. (a) ^{1}H-NMR and (b) ^{13}C-NMR spectra of model compound HBA2HMDA based on HBA and HMDA.

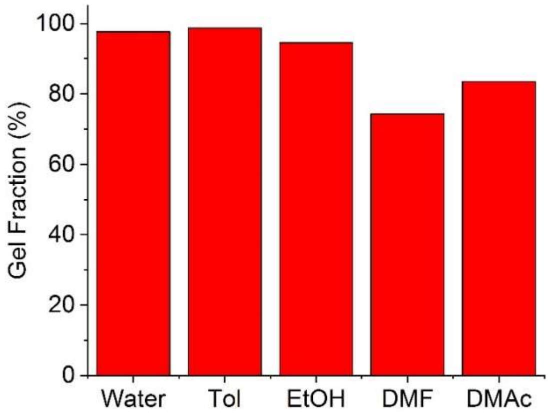

Figure S4. Gel fraction of the epoxy resin cured with Van2HMDA in different solvents.

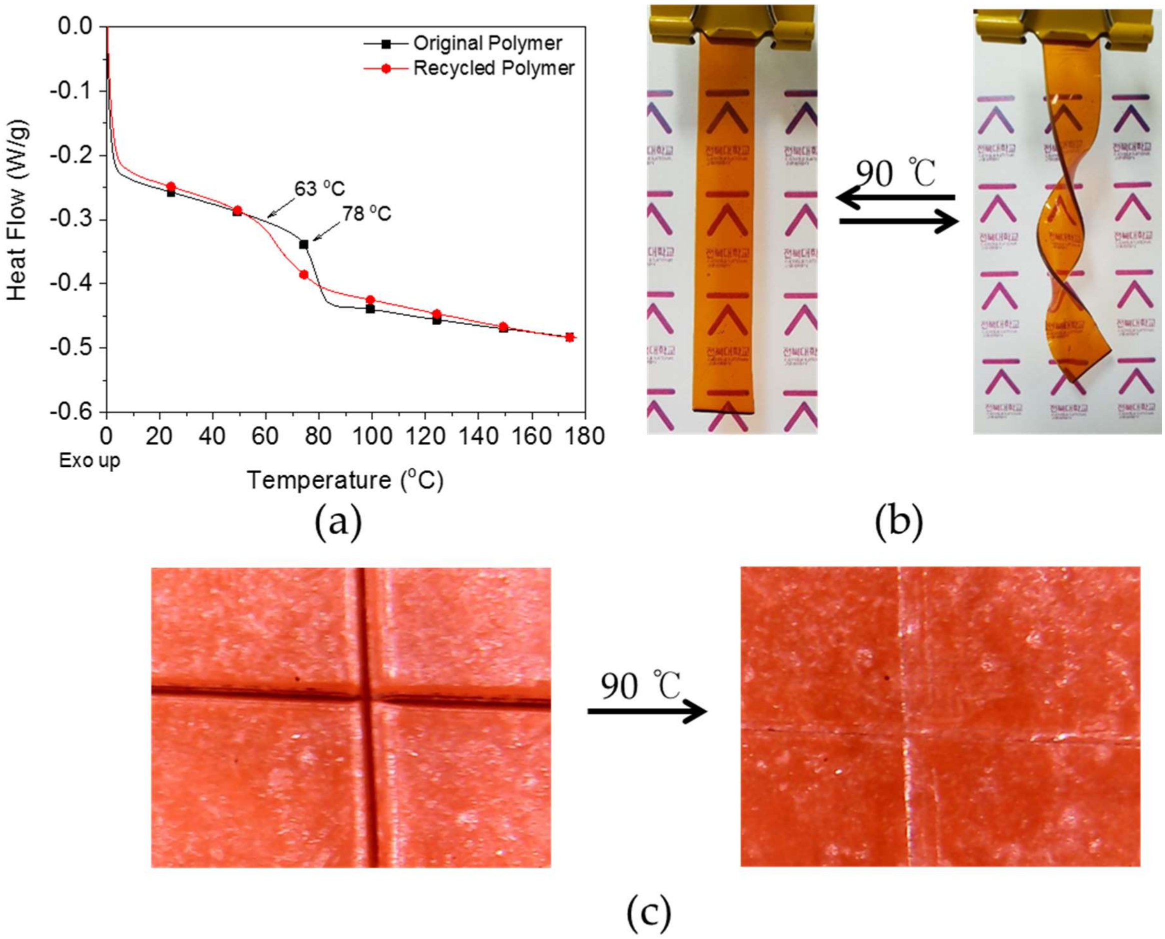

Figure S5. (a) DSC thermalgrams of original polymer and recycled polymer, (b) reshaping ability and (c) thermal healing ability of the recycled polymer.

© 2019 by the authors. Submitted for possible open access publication under the terms and conditions of the Creative Commons Attribution (CC BY) license (http://creativecommons.org/licenses/by/4.0/).
